# Changes of Root Endophytic Bacterial Community Along a Chronosequence of Intensively Managed Lei Bamboo (*Phyllostachys praecox*) Forests in Subtropical China

**DOI:** 10.3390/microorganisms7120616

**Published:** 2019-11-26

**Authors:** Xiaoping Zhang, Zheke Zhong, Xu Gai, Xuhua Du, Fangyuan Bian, Chuanbao Yang, Guibin Gao, Xing Wen

**Affiliations:** China National Bamboo Research Center, Key Laboratory of Resources and Utilization of Bamboo of State Forestry Administration, Hangzhou 310012, China; xiaopingzhang@caf.ac.cn (X.Z.); xugai@caf.ac.cn (X.G.); duxh@cib.ac.cn (X.D.); bianfangyuan@caf.ac.cn (F.B.); chuanbaoy@caf.ac.cn (C.Y.); anshu998@caf.ac.cn (G.G.); wenxing@caf.ac.cn (X.W.)

**Keywords:** bamboo forest, intensive management, plant endophyte, bacterial community

## Abstract

Endophytic bacteria widely exist inside plant tissues and have an important role in plant growth and development and the alleviation of environmental stress. However, little is known about the response of root-associated bacterial endophytes of Lei bamboo (*Phyllostachys praecox*) to intensive management, which is a common management practice for high bamboo shoot production in subtropical China. In this study, we comparatively investigated the root endophytic bacterial community structures in a chronosequence of intensively managed (5a, 10a, 15a, and 20a) and extensively managed plantations (as control, Con). The results showed that endophytic Proteobacteria was the dominant bacterial phylum in the bamboo roots. Intensive management significantly increased (*p* < 0.05) the bacterial observed species and Chao1 (except 5a) indices associated with bamboo roots. The relative abundances of Firmicutes, Bacteroidetes, and Actinobacteria (except 15a) in the intensively managed bamboo roots significantly increased (*p* < 0.05) compared with those in Con, while the relative abundance of Proteobacteria significantly decreased in intensively managed bamboo roots (*p* < 0.05). The phyla Proteobacteria, Actinobacteria, Bacteroidetes, and Firmicutes were the biomarkers in Con, 5a, 15a, and 20a, respectively. Redundancy analysis (RDA) showed that soil alkali-hydrolysable N (AN), available phosphorus (AP), available K (AK), and total organic carbon (TOC) were significantly correlated (*p* < 0.05) with the bacterial community compositions. Our results suggest that the root endophytic microbiome of Lei bamboo was markedly influenced by intensive management practices, and the available nutrient status could be the main driving factor for such shifts. Although heavy fertilization in the intensive management system increased the diversity indices, the rapid changes in root endophyte communities and their relevant functions might indicate a high risk for sustainable management.

## 1. Introduction

The plant-associated microbiome plays an important role in plant health and productivity [1] and has attracted substantial attention from researchers in recent years [2,3,4]. Endophytes are typically defined as microorganisms inhabiting inner plant tissues without harming the host or eliciting strong defense responses [5,6]. Numerous studies have shown that endophytes have beneficial effects on plants, such as providing nutrients, stress tolerance, and pathogen and disease resistance [7,8,9]. Endophytes are also applied for contaminant degradation [10,11] and phytoremediation [12,13]. Due to these important roles, endophytes have immense potential for sustainable agriculture [14,15,16]. Researchers have also found that root-associated endophytic communities are affected by environmental perturbances caused by different agriculture management practices, and the interaction between the changes in endophytes and management practices could be crucial for sustainable management and C sequestration [17,18,19,20].

Lei bamboo (*Phyllostachys praecox*) is an important bamboo species commonly distributed in China with delicious shoots and high economic value to farmers [21,22]. To obtain high yield and economic profits, intensive management, such as heavy fertilization and organic mulching, is a common practice in *P. praecox* plantations [21,23,24]. This technique can provide abundant nutrients to the bamboo ecosystem and alter soil physicochemical and microbial characteristics [25,26]. However, long-term intensive management can cause a series of problems, such as soil acidification and decreased soil organic carbon (SOC) stability [25,27]. Moreover, the mechanisms of the problems caused by intensive management in bamboo forests are not fully understood.

Microorganisms are important aspects of soil quality, nutrient cycling, and plant productivity and health [28,29,30]. Researchers have carried out some studies to estimate the microbiome in bamboo forests under intensive management. According to Zhai et al. [27], intensive management can sharply influence soil bacterial communities, for example, by increasing bacterial abundance and diversity with short-term mulching (less than six years). Li et al. [31] found that intensively managed bamboo plantations can alter the structure and abundance of the fungal community, which is related to the chemical form of SOC. However, the response of bamboo endophytes to intensive management has not been well documented.

In this study, we comparatively investigated the effect of intensive management on the rhizosphere soil properties and root endophytic microbiome of Lei bamboo. The aim was to determine the changes in bamboo endophytes under intensive management and the linkage between shifts in endophytic bacterial communities and changes in soil attributes.

## 2. Materials and Methods 

### 2.1. Experimental Site

This study was carried out at Jingshan township (30°43’ N, 120°30’ E) in Yuhang district, Hangzhou city, Zhejiang Province in southeast China. This region has a subtropical monsoon climate with an annual mean temperature of 15.8 °C and a mean annual precipitation of 1454 mm [27]. The soil is classified as Ferralic Cambisol according to the FAO (Food and Agriculture Organization of the United Nationals) soil classification. The region is a main bamboo shoot production area for *P. praecox*. To obtain high bamboo shoot yield and economic benefits, an intensive management system is usually adopted [27]. For an extensive management system, fertilizer (compound chemical fertilizer, N:P:K = 20:10:15) was applied two times per year (i.e., mid-May and mid-September) at a soil depth of 0–20 cm. The application rate each time was 750–900 kg ha^−1^. In contrast, for the intensive management system, in addition to normal fertilization, organic material mulching and fertilization were applied in November. A mixture of rice husk and rice bran (ratio 9:1) was used as the mulching material. Mulch was applied at a thickness of approximately 30 cm from November to March of the next year. In March of the next year, approximately 70% of the mulching material was removed, and the rest was mixed in the surface soil.

### 2.2. Experimental Design and Sampling

The total study area covered an area of approximately 600 ha of Lei bamboo plantation. The total area is in hilly forestland with a slope less than 10 degrees. The soils are derived from the same sandstone. All bamboo plantations were transformed from natural secondary evergreen broadleaf trees by transplanting mother bamboo plants during the 1980s. To date, more than 80% of plantations are managed intensively because of high economic interest. The intensively managed (with different management age) and extensively managed Lei bamboo plantations were scattered in the whole area. Each managed plantation is about the size of 200 to 1200 m^2^. Based on the filed investigation from Hu et al. [32], they found that one-year-old root of Lei bamboo was in the process of maturing, showing a relative low absorption ability, and 2~3-year-old roots were in a vigorous status, having the strongest absorption ability. Afterwards the root was in a gradual declining stage. Thus, we focused on the 2-year-old roots from bamboo in the current study.

In this area, five replicate plantations for each intensively managed age (5a, 10a, 15a, and 20a) and extensively managed plantations (as control, Con) were randomly selected, with a distance of at least more than 50 m between each other. In each selected plantation, three sampling plots (2.0 m × 2.0 m) were randomly established, and three bamboo plants (2 years old) were selected from each plot for sampling. The root samples were shaken to collect rhizosphere soils and washed with running tap water to remove adhering soil. Then, root tissues were surface sterilized using sterile Millipore water (30 s), 70% (*v*/*v*) ethanol (3 min), sodium hypochlorite solution containing 2.5% active chlorine (5 min), and 70% (*v*/*v*) ethanol (30 s), and finally washed with sterile Millipore water (three times). The final rinse was spread on Luria-Bertani solid medium plates and cultured for 3 d at 28 °C to estimate the surface sterilization. Sterile roots were cut into small fragments, homogenized, and stored in sterile tubes at −80 °C for DNA extraction.

### 2.3. Analysis of Soil Attributes

Soil subsamples were air-dried for chemical parameter analysis. The soil sample pH was measured using a soil-to-water extract at a 1:2.5 (*w*/*v*) ratio. Soil total organic carbon was measured using a total organic carbon (TOC) analyzer (Multi N/C 3100, Analytik Jena AG, Jena, Germany). Soil total N was determined using the Kjeldahl method. Soil alkali-hydrolysable N was determined using a diffusion method according to Lu [33]. Soil available phosphorus was analyzed according to Olsen [33,34]. Soil available K was extracted using 1 mol L^−1^ NH_4_OAc and measured using a flame photometer.

### 2.4. DNA Extraction and High-Throughput Sequencing

Genomic DNA from the root material was extracted using the FastDNA^®^ SPIN kit for soil (MP Biomedical, Santa Anna, CA, USA) following the manufacturer’s instructions. The V3–V4 region of 16S rRNA was amplified using the primer pair 341F (5’-ACTCCTACGGGAGGCAGCAG-3’)/806R (5’-GGACTACHVGGGTWTCTAAT-3’). PCR amplification and Illumina MiSeq PE300 high-throughput sequencing were performed according to the standard protocols of Majorbio Bio-pharm Technology Co., Ltd. (Shanghai, China) and the details were shown in previous studies [35,36,37].

### 2.5. 16S rRNA Gene Sequencing Data Processing

The analysis method for the Illumina sequencing data was performed according to Zhang et al. [4]. Briefly, the pair-end sequences were merged using FLASH [38] and then analyzed in QIIME [39]. The chimeric sequences were identified using Vsearch v2.8.0 [40] against the RDP “Gold” database [41]. Then, the non-chimeric sequences were assigned to operational taxonomic units (OTUs) by applying the open-reference OTU picking protocol in QIIME [39] with default parameters. Sequences belonging to chloroplasts and archaea were removed. Alpha diversity measurements (Shannon, Chao1, and observed species indices) and principal coordinates analysis (PCoA) were performed in QIIME using the rarefied OTU table. Analysis of similarities (ANOSIM) based on Bray-Curtis dissimilarities were also carried out in QIIME. The linear discriminant analysis effect size (LEfSe) method was used to identify the microbial biomarkers [42]. Redundancy analysis (RDA) was performed to determine and visualize the relationship between soil properties and bacterial community composition using the R packages vegan [43] and ggplot2 [44]. Variance inflation factors (VIFs) >10 were removed in the RDA model using the “vif.cca” function. The effects of intensive management on microbial composition and alpha diversity were analyzed using one-way analysis of variance (ANOVA) using IBM-SPSS (version 22.0; Chicago, IL, USA). A value of *p* < 0.05 was considered statistically significant.

## 3. Results

### 3.1. Soil Attributes

Selected rhizosphere soil properties are shown in Table 1. Compared with Con, intensive management significantly increased (*p* < 0.05) the soil total organic carbon (TOC), alkali-hydrolysable N (AN), available phosphorus (AP), and available K (AK). The pH values in Con, 10a, 15a, and 20a significantly decreased (*p* < 0.05) compared with those in 5a. The total N (TN) in 5a, 15a, and 20a was higher (*p* < 0.05) than that in Con and 10a, and no difference (*p* > 0.05) was observed in Con vs. 10a, 20a vs. 5a, and 20a vs. 15a. The C/N ratios in 5a, 15a, and 20a significantly decreased (*p* < 0.05) compared with those in Con, and there was no difference (*p* > 0.05) among 5a, 15a, and 20a and between 10a and Con.

### 3.2. Overall Taxonomic Distribution

After quality filtering and chimera sequence removal, a total of 280,126 bacterial sequences were obtained from the Lei bamboo root samples with a mean value of 11,205 (SD ± 6586) sequences per sample. There were 4516 OTUs with 97% similarity identified from the 25 root samples.

**Figure 1:**
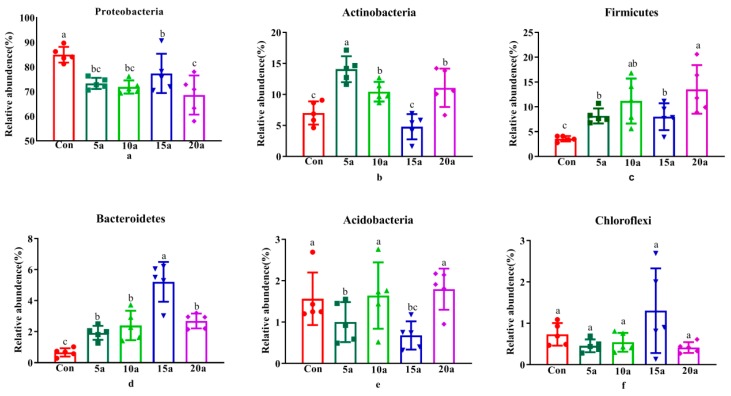
The endophytic microbiome in the Lei bamboo roots at the phylum level. Different letters indicate significant differences among fertilization treatments by one-way ANOVA (LSD, *p* < 0.05). (**a**) Proteobactera; (**b**) Actionobacteria; (**c**) Firmicutes; (**a**) Proteobacteria; (**b**) Actinobacteria; (**c**) Firmicutes; (**d**) Bacteroidetes; (**e**) Acidobacteria; (**f**) Chloroflexi.

The profile of the root endophytic microbiota of Lei bamboo at the phylum level is shown in Figure 1. The bacterial community consisted of six different phyla with an average relative abundance of more than 0.5%, Proteobacteria (75.22%), Actinobacteria (9.48%), Firmicutes (8.89%), Bacteroidetes (2.58%), Acidobacteria (1.34%), and Chloroflexi (0.69%), accounting for more than 98% of the total number of sequences in all samples (Figure 1).

**Figure 2:**
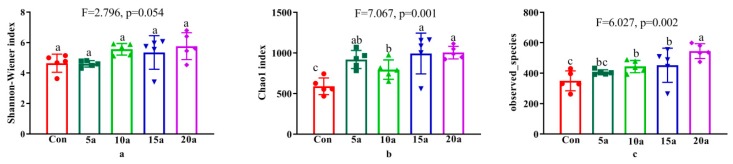
Alpha diversity indices of the bamboo endophytic microbiome in the different intensive management periods and Con. Different letters indicate significant differences among fertilization treatments by one-way ANOVA (LSD, *p* < 0.05). (**a**) Shannon index; (**b**) Chao1 index; (**c**) observed_species index.

The alpha diversity indices (Shannon, Chao1, and observed species indices) are shown in Figure 2. The Shannon, Chao1, and observed species indices increased with increasing durations of intensive management. Compared with Con, more than five years of intensive management significantly increased (*p* < 0.05) the Chao1 and observed species indices but did not significantly change the Shannon index (*p* > 0.05). Chao1 in 5a was also significantly higher (*p* < 0.05) than that in Con.

### 3.3. Shifts in Bacterial Community Structure

The comparison of individual taxa at the phylum level is shown in Figure 1. The results showed that the relative abundances of Firmicutes and Bacteroidetes significantly increased (*p* < 0.05), while the relative abundance of Proteobacteria significantly decreased (*p* < 0.05) in intensive management roots compared with Con roots. The relative abundance of Actinobacteria in 5a, 10a, and 20a was significantly higher (*p* < 0.05) than that in 10a and 15a. The relative abundance of Acidobacteria in 5a and 10a significantly decreased (*p* < 0.05) compared with that in Con, 10a, and 20a.

**Figure 3:**
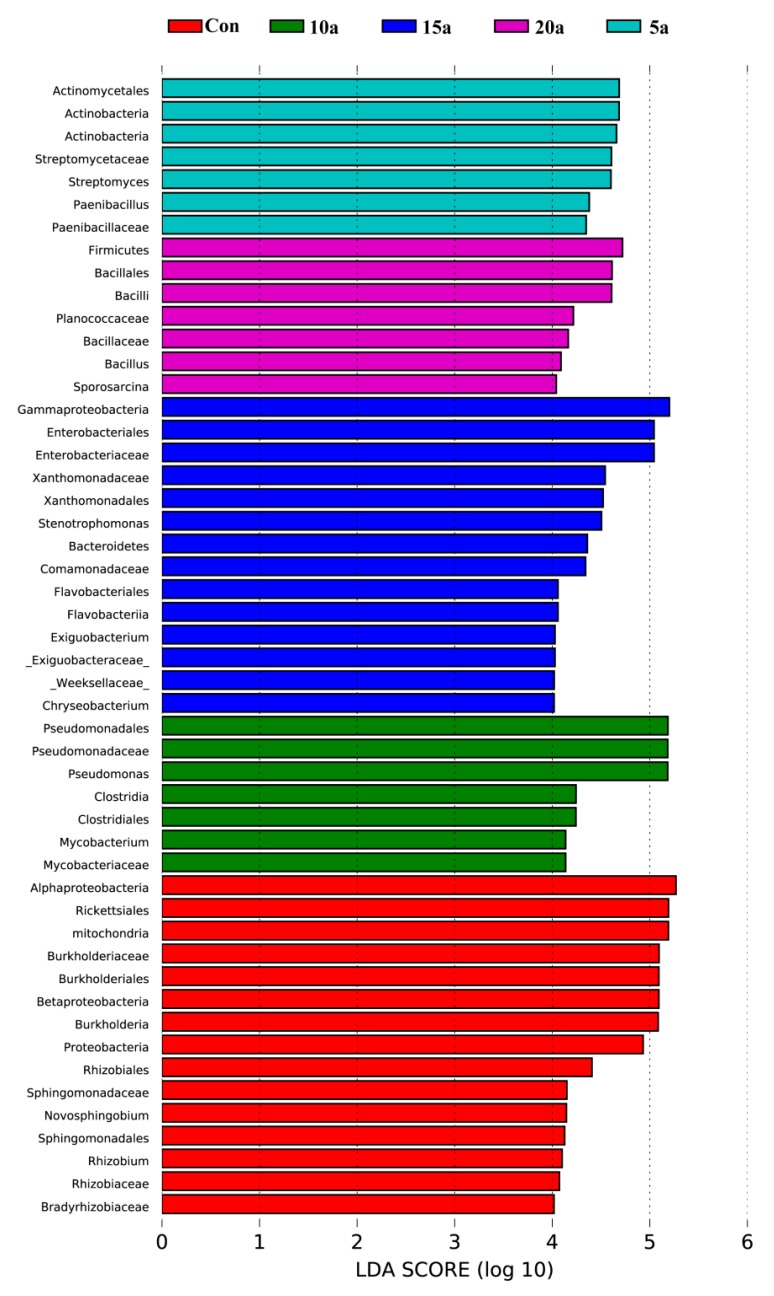
The most differentially abundant taxa were identified by the linear discriminant analysis effect size (LEfSe) method among the different intensive management times. Only taxa meeting an linear discriminant analysis (LDA) significance threshold of >4 are shown.

To further compare the bacterial community compositions among different durations of intensive management, LEfSe was used to analyze the Illumina MiSeq data of the bacterial taxa. There were 50 differentially abundant taxonomic clades with a linear discriminant analysis (LDA) score higher than 4: 15, 7, 7, 14, and 7 clades representing Con, 5a, 10a, 15a, and 20a, respectively (Figure 3). At the phylum level, the bacterial communities of Con, 5a, 15a, and 20a were characterized by an abundance of the phyla Proteobacteria, Actinobacteria, Bacteroidetes, and Firmicutes, respectively. However, there was no biomarker apparent in 10a at the phylum level (LDA score >4). Twelve genus-level biomarkers were identified by LEfSe (LDA score >4). These included three features in Con (*Rhizobium*, *Novosphingobium*, and *Burkholderia*), two features in 5a (*Streptomyces* and *Paenibacillus*), two features in 10a (*Pseudomonas* and *Mycobacterium*), three features in 15a (*Chryseobacterium*, *Stenotrophomonas*, and *Exiguobacterium*), and two features in 20a (*Bacillus* and *Sporosarcina*).

**Figure 4:**
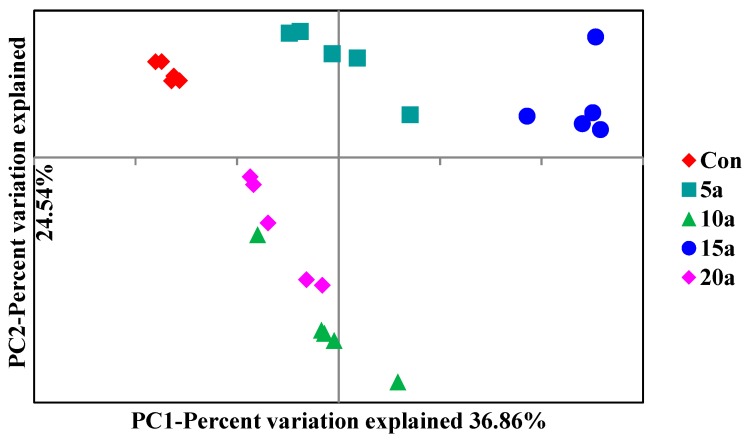
Principal coordinate analysis (PCoA) based on Bray–Curtis distance for the Lei bamboo root endophytic bacterial community structures in the different intensive management periods and Con.

To examine the shift in the root endophytic bacterial community structures in response to intensive management, PCoA was performed based on Bray–Curtis distances (Figure 4). The results revealed that the two principal components explained 61.40% of the variability in the bacterial community, and there was a significant difference in the bacterial community compositions (ANOSIM, *p* = 0.001).

### 3.4. Contribution of Soil Attributes to Bacterial Community Structure

Correlations between soil properties and dominant bacterial phyla (relative abundance >0.5%) were determined using Pearson correlation analysis (Table 2). Soil AN, AP, and AK were negatively correlated with the relative abundance of Proteobacteria and positively correlated with the relative abundances of Firmicutes and Bacteroidetes. The phylum Bacteroidetes was also positively correlated with soil TOC and TN.

**Figure 5:**
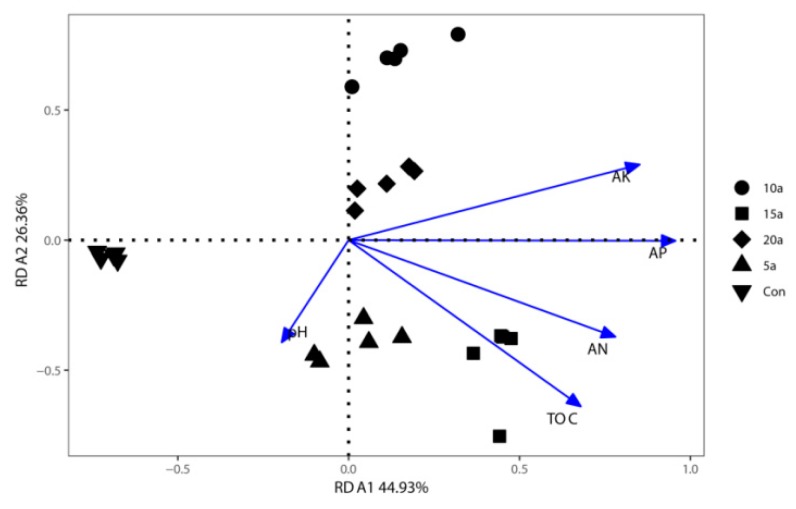
Redundancy analyses of endophytic bacterial community compositions and soil attributes in the Lei bamboo roots.

To identify the soil properties that contributed to shifts in bacterial community structures, RDA was performed to quantify the environmental variables (Figure 5). The RDA analysis had 71.29% of the total variation explained by the first two axes. The results showed that AP (*R*^2^ = 0.79, *p* = 0.001), AK (*R*^2^ = 0.70, *p* = 0.001), AN (*R*^2^ = 0.59, *p* = 0.001), and C/N (*R*^2^ = 0.37, *p* =0.009) were significantly correlated to the bacterial community structures.

## 4. Discussion

According to Gaston [45], alpha diversity is the diversity in population at the local level. Researchers have already showed that alpha diversity of the endophytic microbes can be affected by soil physicochemical properties [18,19,46]. In the current study, we found intensive management had a significantly higher (*p* < 0.05) observed species and Chao1 (except 5a) indices compared with Con, which may be related to the soil attributes in the growing area (Table 1).

The results showed that Proteobacteria was the dominant root endophytic bacteria in Lei bamboo. A similar result was observed in Moso bamboo [47]. Lundberg et al. [48] suggested that soil plays a crucial role in the recruitment of microorganisms by plants. A previous study investigated the soil bacterial communities in the current experimental plots and found that the majority of soil bacteria in Lei bamboo shoots were Proteobacteria [27]. Thus, we speculated that there was also a certain connection between the soil microbiome and root endophytes in bamboo. Members of endophytic Proteobacteria promote plant growth via nitrogen fixation and phosphate solubilization [49,50]. Chen [51] found that long-term intensive management can reduce internal nutrient cycling and be harmful for the growth and regeneration of Lei bamboo. High-throughput sequencing results suggested that intensive management significantly decreased the abundance of the phylum Proteobacteria. The LEfSe analysis also showed that five genus-level biomarkers were identified within the phylum Proteobacteria, including three in Con (*Rhizobium*, *Novosphingobium*, and *Burkholderia*), one in 10a (*Pseudomonas*), and one in 15a (*Stenotrophomonas*), but no genus-level biomarkers within this phylum were selected out in 5a and 20a. Therefore, the relative decrease in Proteobacteria seems to have an impact on plant growth, showing a link between the representation of a bacterial community in the endophytic areas of the root and its proper development. The study also found that intensive management significantly increased the relative abundances of the endophytes Firmicutes, Bacteroidetes, and Actinobacteria (except 15a). Members of the bacterial phylum Firmicutes utilize many carbon sources to produce lactic acid, acetone, butanol, and ethanol [52]. Many Bacteroidetes and Firmicutes strains play an important role in nutrient turnover because they have denitrifying potential [53]. Actinobacteria can also contribute to nutrient uptake in plants [54,55]. The common practice for *P. praecox* forests under intensive management is supplementation with heavy fertilization and surface mulching with organic residues [23], which can increase the extraneous inputs of C and N. Thus, the increase in the abundances of Firmicutes, Bacteroidetes, and Actinobacteria can be involved in the response of the bamboo C and N cycles to the large C and N inputs for intensive management. Additionally, Chen [51] also indicated that long-term intensive management increases the risk of the occurrence and outbreak of leaf-eating insects. Actinobacteria can also inhibit harmful microorganisms and provide a synthesized compound, which indirectly or directly promotes plant growth [56]. Thus, the increase in the abundance of Actinobacteria in roots may be the response of bamboos to the occurrence of pests and diseases. LEfSe analysis also indicated that the bacterial biomarkers in Con, 5a, 15a, and 20a were Proteobacteria, Actinobacteria, Bacteroidetes, and Firmicutes, respectively. Overall, the shifts in microbes were closely related to the changes of soil nutrient status (C and N) and the pH value.

According to Compant et al. [57], physicochemical environmental conditions and nutrition characteristics are key factors contributing to the bacterial community compositions in soil and plants. Zhao et al. [58] showed that the relative abundance of Proteobacteria was positively correlated with soil AN and AK, while Bacteroidetes was positively correlated with soil AK and TP and negatively correlated with soil organic matter and TN. Zhao et al. [59] found that the relative abundances of Gammaproteobacteria and Bacteroidetes were positively correlated with soil pH, AK, and the C/N ratio and negatively correlated with AP and TN (only Bacteroidetes). A study from Cui et al. [60] indicated that the relative abundance of Firmicutes was negatively correlated with pH and positively correlated with AP in the bulk and rhizosphere soil of maize. In the current study, the relative abundances of Proteobacteria (negative), Firmicutes (positive), and Bacteroidetes (positive) were significantly correlated with soil AN, AP, and AK. We also found that soil TOC and AN were also positively correlated with Bacteroidetes. Thus, soil nutrient availability has higher correlations (positive or negative) with the majority of the endophytic bacterial taxa in Lei bamboo. These results are consistent with the findings of Robinson et al. [17], who found that wheat endophytic bacterial community compositions can be shaped by soil nutrient availability.

Recent studies have evaluated the effect of management regimes on bacterial endophytes in plant species, such as rice [19], wheat [17], maize [61], and grass [62]. The results of the current study also indicated that intensive management markedly influenced the Lei bamboo root endophyte microbiome. Ren et al. [63] found that endophytic bacterial communities of Jingbai pear trees were significantly associated with soil properties, including the pH and N and C contents. According to Robinson et al. [17], soil nutrient availability can affect the wheat endophytic bacterial community through the plant growth response. Xia et al. [64] showed that soil pH plays a role in endophyte community diversity in switchgrass. In the current study, endophytic bacterial community structure was significantly correlated with soil AN, AP, AK, and C/N. Thus, shifts in soil properties contribute to changes in the endophytic bacterial community caused by intensive management.

## 5. Conclusions

This study revealed that bamboo roots harbored rich and diverse bacterial endophytes. Intensive management markedly changed the endophytic bacterial communities in two-year-old root from Lei bamboo. The alteration in the endophytic bacterial taxa is highly related to plant nutrient status, which fluctuated with the intensive management time. The study also enhances our understanding of the interaction between management practices and the bamboo-associated microbiome and helps us to develop a more sustainable management way for bamboo plantations.

## Figures and Tables

**Table 1:** Selected property of the rhizosphere soils from Lei bamboo in different intensive management times.

	pH	TOC (g/kg)	AN (g/kg)	AP (g/kg)	AK (mg/kg)
Con	4.55 ± 0.25 b	25.66 ± 0.92 c	159.12 ± 25.25 d	45.81 ± 12.06 c	114.32 ± 16.64 b
5a	5.35 ± 0.59 a	44.51 ± 4.47 b	409.49 ± 80.07 b	159.90 ± 14.31 ab	240.60 ± 100.31 a
10a	4.23 ± 0.32 b	26.59 ± 3.18 c	241.56 ± 58.52 c	145.52 ± 16.62 b	303.20 ± 87.01 a
15a	4.21 ± 0.19 b	59.04 ± 12.80 a	417.50 ± 42.97 ab	163.41 ± 13.94 a	278.99 ± 41.66 a
20a	4.56 ± 0.41 b	54.37 ± 4.22 a	480.82 ± 26.43 a	154.94 ± 5.15 ab	311.98 ± 77.16 a

Notes: 5a, 10a, 15a, and 20a, mulching for 5, 10, 15, and 20 years; Con, extensively managed bamboo plantations; TOC, total organic carbon; AN, alkali-hydrolysable N; AP, available phosphorus; AK, soil available K. Different letters indicate significant differences among fertilization treatments by one-way ANOVA (Least-Significant Difference (LSD), *p* < 0.05).

**Table 2:** Correlation analysis of the dominant phyla and soil attributes.

	pH	TOC	AN	AP	AK
Proteobacteria	0.012	−0.266	−0.472 *	−0.583 **	−0.477 *
Actinobacteria	0.341	−0.118	0.178	0.295	0.095
Firmicutes	−0.064	0.308	0.497 *	0.507 **	0.544 **
Bacteroidetes	−0.318	0.624 **	0.474 *	0.613 **	0.439 *
Acidobacteria	−0.288	−0.347	−0.261	−0.208	−0.212
Chloroflexi	−0.182	0.148	−0.048	−0.043	−0.001

TOC, total organic carbon; AN, alkali-hydrolysable N; AP, available phosphorus; AK, soil available K; * *p* < 0.05; ** *p* < 0.01.
